# Expanding pathways to clinical and translational research training with stackable microcredentials: A pilot study

**DOI:** 10.1017/cts.2024.601

**Published:** 2024-09-30

**Authors:** Marie K. Norman, Thomas R. Radomski, Colleen A. Mayowski, MaLinda Zimmerman-Cooney, Isabel Crevasse, Doris M. Rubio

**Affiliations:** 1 Institute for Clinical Research Education, University of Pittsburgh School of Medicine, Pittsburgh, PA, USA; 2 Division of General Internal Medicine, University of Pittsburgh School of Medicine, Pittsburgh, PA, USA

**Keywords:** Distance learning, microcredentials, online learning, training, workforce development

## Abstract

**Introduction:**

The proportion of physician-investigators involved in biomedical research is shrinking even as the need for high-quality, interdisciplinary research is growing. Building the physician-investigator workforce is thus a pressing concern. Flexible, “light-weight” training modalities can help busy physician-investigators prepare for key stages of the research life cycle and personalize their learning to their own needs. Such training can also support researchers from diverse backgrounds and lighten the work of mentors.

**Materials and Methods:**

The University of Pittsburgh’s Institute for Clinical Research Education designed the Stackables Microcredentials in Clinical and Translational Research (Stackables) program to provide flexible, online training to supplement and enhance formal training programs. This training utilizes a self-paced, just-in-time format along with an interactive, storytelling approach to sustain learner engagement. Learners earn badges for completing modules and certificates for completing “stacks” in key competency areas. In this paper, we describe the genesis and development of the Stackables program and report the results of a pilot study in which we evaluated changes in confidence in key skill areas from pretest to posttest, as well as engagement and perceived effectiveness.

**Results:**

Our Stackables pilot study showed statistically significant gains in learner confidence in all skill areas from pretest to posttest. Pilot participants reported that the module generated high levels of engagement and enhanced their skills, knowledge, and interest in the subject.

**Conclusions:**

Stackables provide an important complement to formal coursework by focusing on discrete skill areas and allowing learners to access the training they need when they need it.

## Introduction

The declining proportion of physician-investigators in biomedical research has been a cause for concern since Wyngaarden first referred to physician-investigators as “an endangered species” in 1979 [1–4]. Indeed, the percentage of physician-scientists in the workforce fell from 4.75% in the 1980s to 1.5% in the 2020s [5,6]. With biomedical research expanding, there is a critical need to ensure that physicians enter and remain in the research workforce [7].

Addressing this problem will require a multi-pronged approach that includes new forms of training [8,9]. It is our contention that well-designed, flexible, self-paced learning options can help to address the needs of physician-investigators, not to mention other learners [10–17]. Without replacing formal training programs, these learning modalities can allow physician-investigators to access training when and where they need it, targeting learning to their specific goals and experience levels and accommodating their all-too-busy schedules. By making difficult concepts and skills more approachable, moreover, these training approaches can also create valuable on-ramps for other learners, including those from diverse disciplinary and cultural backgrounds. They can give undergraduates and medical students a taste of clinical research before committing to a research career. Finally, they can relieve pressure on over-taxed mentors by providing short, targeted, skill-focused trainings to share with mentees.

The Stackables Microcredentials in Clinical and Translational Research (Stackables) program at the University of Pittsburgh’s Institute for Clinical Research Education (ICRE) is a new program, designed to make clinical and translational research training more accessible, convenient, and relevant to busy professionals. In this paper, we describe the development of the program and evaluate the results of a pilot study.

## Background

Physician-investigators bring a unique and valuable perspective to health research. Because of their direct, clinical experience, they are particularly well positioned both to identify important research questions and to bring evidence-based practices back into clinical care [18]. Unfortunately, the proportion of physicians pursuing research careers has been in decline for four decades [7,19].

Despite encouraging signs in the early 2000s when the National Institutes of Health (NIH) developed new programs to attract more clinicians to research [7], NIH budgets have failed to keep pace with rising research costs and physician participation in research has again dropped [8]. An aging workforce [9] and burnout among physicians [20–22] have exacerbated the problem. Clinical and translational research may be particularly affected by the attrition of physician-investigators. Indeed, a recent study showed that over half of physician-investigators involved with clinical and translational research left the field after a single trial [23]. The problem, moreover, is not limited to the USA [2,24,25].

A number of reasons have been cited for this decline, including the intensive time required for clinical training, lack of research opportunities, educational debt, the difficulty of balancing clinical and research duties, competition for research funding, and lack of leadership training [9]. The difficulty of managing work-life balance was cited as a primary reason physician-investigators have left clinical and translational research in particular [23]. Added challenges face women and people of color [9,26].

We believe that more varied and flexible types of training are needed, training that supports researchers at specific stages of the research cycle (e.g., grant writing, data collection, data analysis, manuscript writing) and can be personalized to the learner’s goals and experience level. Such training should target the needs of a broad range of learners, including those who have families and demanding jobs, who lack the time to engage in formal course work, do not live in proximity to institutions where clinical and translational research degrees are offered, need very specific training but not an entire degree, cannot wait until a course is offered to learn what they need to know, and/or are still exploring the field and are not ready to commit to a research career.

### Unbundling and microcredentials

Thought leaders in higher education have long called for academia to “unbundle the curriculum” [27,28] by disaggregating traditional degree programs into smaller, lighter, and generally less expensive components that learners can then reassemble into personalized learning paths [11,13–16]. Unbundling represents a response to changes in the educational landscape, including (a) a growing gap between skills needed in the workforce and the skills graduates possess [29]; (b) increasing enrollment of adults seeking specific, practical skills [30]; (c) rapid, technology-driven changes in the workplace, for example, new areas of medical research that demand a quickly updatable, adaptive curriculum [29,31]; (d) efforts to address the rising cost of higher education [32]; and (e) the widespread availability of technology-enabled learning modalities (e.g., remote, self-paced) that extend educational access to new student populations [29].

The unbundling movement has led to a focus on microcredentials: collections of short courses that are more practical and less theoretical than standard courses. Microcredentials generally utilize digital technologies and online, asynchronous modalities [27,33,34], and learners earn microcredentials, such as badges and certificates, rather than full degrees [34–36]. Microcredentials have a number of advantages. Because they are smaller and lighter than full-length courses they take less time to develop. Thus, they lend themselves to rapidly changing and newly emerging subject areas. Their smaller size and practical approach make them appealing to busy adult learners [37]. Moreover, microcredentials have been linked to positive outcomes, including achievement, confidence, and engagement [34–36,38–40].

### Asynchronous, self-paced learning

With “asynchronous learning,” learners are engaged with learning materials but are never required to be in the same place (physical or virtual) at the same time. Asynchronous learning has the distinct advantage of allowing learners to engage with instruction entirely on their own time (e.g., at night after work, when the kids are playing.) It also allows them to move at their own pace: learners who require more time can take it while learners who do not can jump ahead. While requiring an initial investment in course development, asynchronous learning can be more easily scaled than synchronous learning and can thus help to reduce costs for learners [41,42]. Motivation can be attenuated in asynchronous learning, where learners lack a sense of connection and accountability to instructors and/or other learners [43,44]. However, careful design strategies can help to overcome the motivational challenges, including the following:

- **Enlisting multimedia design principles to reduce cognitive load**. Cognitive tasks such as navigating poorly designed interfaces or confusing instructions sap cognitive resources and do not contribute to learning. Following multimedia design principles can help to reduce extraneous cognitive load [33,45,46].
- **Making the learning experience enjoyable**. Beautiful visual design of instructional materials is an under-appreciated factor in learning. It increases both learner engagement and persistence by enhancing the enjoyment of the experience [37,47].
- **Incorporating interactivity to ensure engagement** [48–54]. Years of research show that we learn better when we actively engage with ideas rather than absorbing them passively.
- **Using storytelling to generate a sense of real-world relevance and value**. Incorporating stories into learning grabs and focuses attention, which is critical to learning [55–57].

We have enlisted these research-based design principles in the development of Stackables to ensure that learners receive the benefits of asynchronous, self-paced learning without the drawbacks.

## Methods

### Overview and target audience

ICRE Stackables are self-paced, online modules that can be mixed and matched to build competencies. They were intended to supplement rather than replace traditional degree programs by (a) providing additional and more flexible avenues into clinical and translational training; (b) broadening access for learners who cannot participate in degree programs, whether because of time constraints, financial barriers, or simply the locality of such programs; and (c) addressing the needs of learners who are seeking to acquire or brush up on very specific skills and do not require a full degree or certificate program. Table 1 describes the characteristics Stackables were designed to have.

**Table 1.** Module characteristics and goals

Characteristic	Goals
Skill-focused	Each module focuses on a specific skill area and work product (e.g., writing a discussion section, developing a qualitative codebook) and provides steps, models, and examples to help learners develop the process awareness and analytical skills necessary for the target task.
Bite-sized	Each module takes no more than one hour to complete and corresponds roughly to the material covered in an average class session. The smaller package allows learners to fit learning opportunities around busy schedules.
Just-in-time	Modules are available precisely when learners need them, for example when writing a grant proposal or responding to reviewer comments on a manuscript, so that they do not need to wait for courses or seminars to be offered.
Personalized	Modules disaggregate curriculum into small pieces, which allows learners to access only the training they want or need and permits the research-curious to “test the waters” without making a significant commitment.

### Organization and format

ICRE Stackables are organized into “stacks,” each defined by a topic (e.g., Implementation Science, Clinical Research Fundamentals). Each stack comprises a set of modules focused on a discrete skill set within the topic area. The qualitative research methods stack, for instance, includes modules such as writing a qualitative interview guide, conducting a qualitative interview, developing a qualitative codebook, and coding qualitative data. Learners can earn badges by completing individual modules or complete three modules in the stack to earn a certificate of completion.

All the modules follow a similar format to ensure structural consistency. This consistency helps learners know what to expect and how to navigate the module while using storytelling, active learning, graphic design principles, and multimedia design principles to keep learners engaged. Each module includes:

- **A research case study**: The case, which evolves over the three sections of the module, features a protagonist at a recognizable research stage (e.g., defining a research question, preparing to write a survey).
- **A set of three branching scenarios**: Each scenario is built around common “rookie” mistakes and highlights key decision points, choices, and consequences.
- **Interactive didactic content**: Didactics grow out of the case and scenario, filling in information our protagonist needs to navigate the tasks they are facing. Didactic portions are written in a conversational, accessible style and feature light interactivity (e.g., diagrams with hot spots, clickable timelines, drag-and-drop exercises).
- **Knowledge-check questions**: Each section ends with knowledge-check questions that assess learner comprehension. Feedback on answers provides additional nuance.

While we incorporated multimedia into the modules, we intentionally did not rely on video. There were several reasons for this. First, text is easier to revise and update than video [58]. Second, learners can interact with modules anywhere without worrying about audio or headphones.

We wanted the learner’s experience in this program to feel intuitive and inviting, so we chose tools and platforms we felt could facilitate a seamless, enjoyable experience. We selected Rise 360™ for content authoring because it is clean, modern, and aesthetically pleasing and includes a variety of features to facilitate interactivity. We chose LearnDash™ (a WordPress product) to host the modules and serve as our learning management system. LearnDash manages enrollments and payment, tracks learners’ progress in an analytics dashboard, and issues badges and certificates.

### Module development

Modules were developed by faculty subject matter experts (SMEs) in collaboration with the Innovative Design for Education and Assessment (IDEA) Lab at the University of Pittsburgh’s ICRE. The IDEA Lab leads educational innovation at the ICRE and has deep expertise in the learning sciences, online education, instructional design, graphic design, and video production. In the development of modules, SMEs provided content expertise and helped the IDEA Lab develop “scripts” (text versions of cases, branching narratives, didactic content, questions, and feedback), which the IDEA Lab then built out as multimedia, interactive modules using the Rise 360 authoring tool. The process of module development was highly iterative, involving multiple rounds of discussion and revision.

### Assessment

In 2023, we conducted a pilot study of the Stackables using a survey-based, pre-/posttest design. We recruited study participants via an email distribution list of current and former students in ICRE programs, which included certificate, masters, and PhD students in clinical research and medical education or alumni of these programs. Many of them were physicians and some were faculty. We offered participants access to one of the Stackables modules in exchange for completion of the module and a brief survey at the beginning and the end. We opted to include a single module rather than multiple modules to limit the burden on respondents and ensure that participants were all evaluating the same thing. We chose the module *Recruiting for Qualitative Studies* because we thought it might have crossover value to both qualitative and quantitative researchers.

We sent the module link and password to the 25 people who responded affirmatively to our recruitment email, specifying that they would have three weeks to complete the pretest, module, and posttest (pre- and posttests were embedded at the beginning and end of the module itself). On the pretest, participants were asked to describe their previous exposure to qualitative research on a 5-point Likert scale (a lot, a little, neutral, not much, none at all) and rate their confidence in key skill areas (not at all confident, not very confident, neutral, confident, very confident).

On the posttest, participants were asked to rate their confidence in the same skill areas as the pretest using the same scale. They were also asked a series of questions about the extent to which the module contributed to their learning, interest, and skill development, kept them engaged, and compared them to other learning experiences. They were also given the opportunity to provide additional feedback on two open-ended questions included as well: “How can we improve this module?” and “Please share any other thoughts or comments you have about the module.” (See survey questions and scales in Supplementary Materials 1.)

Descriptive analysis was first conducted to measure the frequency and mean of response ratings, with the responses pooled for pre- and posttest group comparison due to the unequal sample size. Statistical significance between pre- and posttest confidence responses was measured using a two-tailed *t*-test.

## Results

A total of 19 participants completed the pretest and 17 of the 19 completed the posttest. On the pretest, 84% of respondents indicated having “a little” experience with qualitative research prior to taking the module.

**Figure 1.**
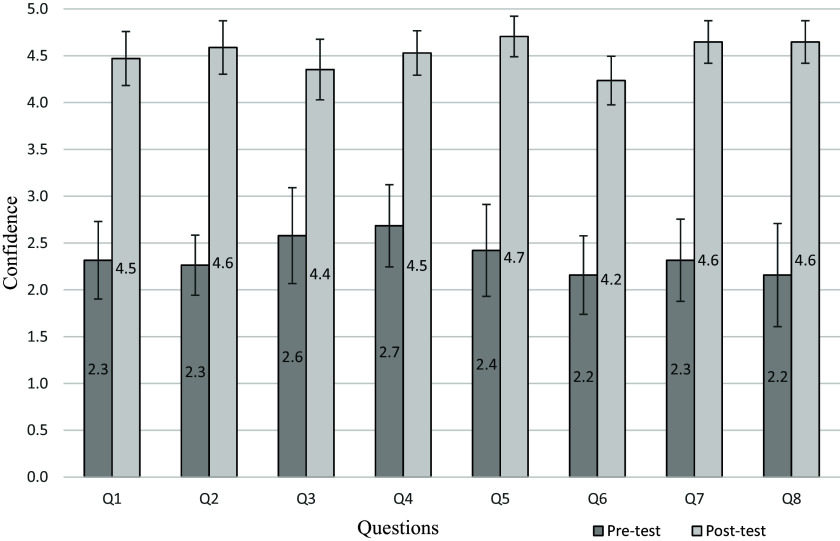
Average self-reported confidence in key skill areas from pretest (*n* = 19) to posttest (*n* = 17). All items showed a statistically significant increase as calculated in two-tailed *t*-tests.

We saw statistically significant improvement in confidence ratings from pretest to posttest with *t*-tests showing a *p*-value of 0.001 between all pre- and posttest confidence scores. On the pretest, participants reported low levels of confidence in all skill areas (see Figure 1) with scores between 1.5 and 2 in all areas, indicating Likert scale values of “not at all confident” and “not very confident.” The lowest confidence scores were in three skill areas: (1) selecting the best recruitment method(s) for a given study, (2) identifying which recruitment materials to include in your Institutional Review Board (IRB) proposal, and (3) identifying key issues to consider when developing a recruitment plan for a qualitative study.

In the posttest, all scores had moved to a range between 3.5 and 5, with 88% of respondents indicating that they now felt “confident” or “very confident” (50%) in six of the eight categories (see Figure 1). The smallest increase in confidence was on Question 1, which asked how confident respondents felt in “Identifying the key issues to consider when developing a recruitment plan for a qualitative study.” This item showed a confidence gain of 1.3 points.

In addition, when asked the extent to which module elements (e.g., case study, branching narrative, didactic content, knowledge-check questions) contributed to their learning, 84% of respondents rated all module elements “above average” or “high” on a 5-point Likert scale (low, below average, neutral, above average, high.) Ninety-four percent of participants reported that the module contributed to their learning and kept them engaged. Additionally, 65% of respondents said that the module was at minimum “better” than traditional classroom learning, with 35% rating it “much better.” Eighty-eight percent stated they would recommend this module to a friend and be willing to take another module similar in design. Lastly, 100% of respondents felt the module enhanced their understanding of the topic, 76% stated that it increased their interest in the topic, and 94% reported that the module enhanced their skills. Finally, 47% felt that they completed the module well within the time estimate.

Participants left a total of eight comments in the “how can we improve this module?” field and eight in the “thoughts and comments” field. Suggestions for improvement included everything from easily modifiable elements (changes to fonts, background colors) to somewhat more involved changes (add more case studies and multimedia) to elements that could not be addressed without jeopardizing scalability (e.g., making this a hybrid course with human interaction).

Responses to the general comments (Table 2) question clustered around two general themes: learner engagement and content level. Participants described feeling engaged and identified design elements (case study, knowledge-check questions) that contributed to their engagement. They also seemed to express appreciation that the modules were introductory and provided a baseline understanding of the topic.

**Table 2.** Representative responses (each bullet corresponds to a different respondent)

Learner engagement	Content level
Really engaging and thoughtful approach. I liked the way I could go back and look at the wrong answers. I liked the idea of having an evolving case study. The only thing missing is the mood music. :) I felt like the graphics were all pertinent, not just filler. I liked that it was text based rather than audiovisual.	I think these modules are good introductory materials to consider for researchers who are learning about qualitative methods. I am not sure I would say my skill increased, but rather my knowledge base. For skill to increase, I would have to put these recruitment methods into practice. It was very helpful for the basics of community qualitative research. I am interested in this kind of research on local Turkish immigrants as I am very close to them for their health-related questions.

There were slightly mixed reactions to the module format, which uses text, images, and interactive elements. One participant suggested we incorporate more audio/video components, while another appreciated that the module did *not* use audio/video components. Although the majority of respondents (65%) reported in Likert scale questions that the module was “better” or “much better” than traditional classroom learning, one person questioned in the comments whether the modules were a substitute for traditional, synchronous courses.

## Discussion

This study evaluated the capacity of ICRE Stackables to provide effective clinical and translational research training in a flexible, personalized, engaging format. Our pilot test of the Stackables showed strong results. Learners reported that the modules increased their interest, skills, and knowledge and expressed interest in taking more modules of similar design. They also reported statistically significant increases in confidence in all skill areas from pretest to posttest. The area that showed the most modest increase in confidence (“Identifying the key issues to consider when developing a recruitment plan for a qualitative study”) may be due to learners gaining a more realistic understanding of the complexity of recruitment by taking the module. Most encouraging to us, learners found the design of the modules engaging.

The success of this pilot suggests to us that self-paced, asynchronous learning can be effective for busy, adult learners *if* it is designed carefully to maximize engagement and provide skill-based, just-in-time training in bite-sized portions. At the same time, participant comments suggest that care must be taken to set expectations about the level of the courses (beginner) and their purpose (not to replace for-credit courses).

The decline in physician-investigators, combined with the need to provide easier on-ramps for learners from diverse disciplinary and cultural backgrounds, presents us with a number of challenges: How can we forge new pathways into research careers and provide training that meets learners where they are? And how can we enhance existing research training programs by offering flexible, personalized training precisely when and where it is needed? We see this program playing a valuable role in addressing these challenges by expanding the types and modalities of training available to physician-investigators and other members – or potential members – of the clinical and translational workforce. We see Stackables as a valuable complement to established degree and training programs, with the potential to help learners who are not well-served or sufficiently served by existing programs.

Because they are bite-sized and modular, moreover, Stackables may also help to address emerging research priorities. For instance, in 2022, the National Center for Advancing Translational Science (NCATS) announced a new focus on “understanding the scientific and operational principles underlying each step of the translational process” [59], overcoming long-standing barriers, and increasing the speed at which research is translated into practice and policy. We believe Stackables can play a key role in shortening the time to translation by providing a more timely and efficient way for researchers to gain the skills and knowledge they need. Using Stackables, physician-investigators and other researchers can quickly learn about an unfamiliar methodological approach before writing a grant, refresh their skills before beginning a project, access just-in-time training at specific project stages (e.g., before beginning qualitative interviews), or address a skill or knowledge gap. Researchers from different disciplines or cultures can familiarize themselves with the conventions of clinical research in the USA without embarrassment. Moreover, over-busy mentors can direct mentees to skill-specific training, a welcome alternative to having to teach these skills themselves or asking mentees to wait for the appropriate for-credit course to be offered. Moreover, by providing a lower-commitment, more accessible, more personalized on-ramp into research, Stackables can help to attract new people to the field.

There are, of course, limitations to using a microcredentialing approach. Critics of microcredentialing question whether employers will accept microcredentials in lieu of degrees [60]. They also wonder if, by disaggregating curricula, microcredential programs lose educational coherence [61]. Both these critiques are valid. However, we do not see them as major obstacles in our context. Many of our target learners are physicians, who already possess advanced degrees and hold professional positions. Their primary motivations are to acquire skills rather than credentials, which makes concerns about employer acceptance of microcredentials somewhat less salient. To the second point, Stackables are designed specifically for learners who need targeted training to facilitate certain tasks (e.g., writing a grant proposal, developing a qualitative codebook.) In such cases, learners generally know what they need to learn and are less reliant on a larger curriculum. That being said, the coherence of a formal curriculum could be provided in part by a pre-assessment that recommends modules or stacks based on career stage, research focus, and prior experience. There were also limitations to our pilot assessment. We only tested one module and did so with learners at a single institution. More data will need to be collected as new stacks are added and disseminated to different populations of learners.

To that point, our plan is to launch the Stackables in late 2024, incorporating our first three stacks: foundations of clinical research, qualitative research methods, and scientific writing. As stacks are disseminated, we will collect data to improve existing modules, the design of subsequent stacks, and our dissemination strategy. Our plan is to add stacks until we have enough modules to populate a Stackables Marketplace, which learners will be able to browse and search to find stacks and modules of interest. We also plan to collaborate with other institutions to develop stacks in emerging skill areas and to target new audiences of learners, such as research staff.

## Conclusion

We have seen a pressing need for more flexible learning options to address the specific training needs of physician-investigators, as well as those of an increasingly diverse research community. Stackables unbundle the traditional curriculum into engaging, bite-sized, self-paced learning experiences that are accessible to researchers when and where they need them and both supplement and enhance traditional training programs. They capitalize on the flexibility of asynchronous learning while also sustaining learner engagement by using storytelling and interaction. Stackables offer a valuable model for helping to build and sustain the careers of physician-investigators while creating new on-ramps and supports for researchers from diverse disciplinary and cultural backgrounds.

## Supporting information

Norman et al. supplementary material Norman et al. supplementary material

## Supplementary material

The supplementary material for this article can be found at https://doi.org/10.1017/cts.2024.601.
