# Supplementary material for: Expanding pathways to clinical and translational research training with stackable microcredentials: A pilot study
Source: J Clin Transl Sci. 2024 Sep 30;8(1):e138. doi: 10.1017/cts.2024.601 (PMC11523017; doi:10.1017/cts.2024.601)
Supplement: Norman et al. supplementary material [file S2059866124006010sup001.docx]

# Supplementary Material 1

## Pilot Assessment Survey Pre-Test Questions

- Please rate your confidence in the following areas [Likert: Not very confident, not confident, neutral, confident, very confident.]
  - Identifying the key issues to consider when developing a recruitment plan for a qualitative study
  - Identifying at least three strategies to lower barriers to participation in a qualitative study
  - Identifying a range of recruitment challenges unique to vulnerable populations
  - Identifying strategies for building trust when recruiting from vulnerable populations
  - Identifying at least three recruitment methods for qualitative studies
  - Selecting the best recruitment method(s) for a given study.
  - Identifying the specific information you must include in recruitment materials.
  - Identifying which recruitment materials to include in your IRB proposal.
- How much exposure have you had to qualitative research previously? [Likert: A lot, a little, not much, none at all]

## Pilot Assessment Survey Pre-Test Questions

- Please rate your confidence in the following areas [Likert: Not very confident, not confident, neutral, confident, very confident.]
  - Identifying the key issues to consider when developing a recruitment plan for a qualitative study
  - Identifying at least three strategies to lower barriers to participation in a qualitative study
  - Identifying a range of recruitment challenges unique to vulnerable populations
  - Identifying strategies for building trust when recruiting from vulnerable populations
  - Identifying at least three recruitment methods for qualitative studies
  - Selecting the best recruitment method(s) for a given study.
  - Identifying the specific information you must include in recruitment materials.
  - Identifying which recruitment materials to include in your IRB proposal.
- To what extent did this module contribute to your learning? [Likert: very much, somewhat, neutral, not very much, not at all]
  - Explain
- To what extent did the module keep you engaged? [Likert: not at all engaged, somewhat disengaged, neutral, somewhat engaged, very engaged]
  - Explain
- How would you compare this type of self-paced learning to traditional classroom learning? [Likert: Much better, better, equivalent, worse, much worse]
- How can we improve this module? [Text box]
- How likely would you be to recommend this module to a friend? [Likert: very likely, likely, neutral, unlikely, very unlikely]
- How likely would you be to take another module with a similar design? [Likert: very likely, likely, neutral, unlikely, very unlikely]
  - Unlikely and very unlikely [Skip logic 🡪 Please explain]
- How would you rate the accuracy of the following statements? [Likert: Very true, somewhat true, neutral, somewhat untrue, very untrue]
  - This module enhanced my understanding of the topic
  - This module increased my interest in the topic
  - This module enhanced my skills
  - Please explain any of your ratings above. [Text box]
- How would you rate the value of the following module elements to your learning? [Likert: Very high, above average, average, below average, very low]
  - Evolving case study (i.e., Alma’s story)
  - Branching narratives (i.e., when you are asked to help Alma make a choice)
  - Didactic elements (i.e., information about study recruitment)
  - Examples (i.e., sample recruitment emails and phone script, sample poster)
  - Knowledge check questions
- How accurate were the time estimates for completing this module?
  - Accurate
  - Not accurate
    - [Skip logic 🡪 Please explain]
- Please share any other thoughts or comments you have about the module [text box]
